# Gender differences in the learning and teaching of surgery: a literature review

**DOI:** 10.5116/ijme.5380.ca6b

**Published:** 2014-06-15

**Authors:** Carmen Mesas Burgos, Anna Josephson

**Affiliations:** 1 Division of Pediatric Surgery, Department of Women’s and Children’s Health, Karolinska Institutet, Sweden; 2 Department of Neuroscience, Karolinska Institutet, Sweden

**Keywords:** Gender, surgery, medical education, surgical education, undergraduate, teaching and learning

## Abstract

**Objectives:**

To explore evidence concerning gender differences in teaching and learning in surgery to guide future initiatives.

**Methods:**

This systematic review was conducted searching in the following electronic databases: MEDLINE, EMBASE, CINAHL, PsycINFO, ERIC, Web of Science, Scopus and PubMed. All studies related to gender differences in surgical education, teaching or learning of surgery at an undergraduate level were included. Data was extracted and critically appraised. Gender differences in learning, teaching, skills acquisition, perceptions and attitudes, interest on surgery, personality and factors influencing interest in surgical careers were differentiated.

**Results:**

There is an underrepresentation of women in surgical academia, due to lack of role models and gender awareness. It is not clear whether or not gender itself is a factor that affects the learning of surgical tasks. Female students pursuing a surgical career had experienced sexual harassment and gender discrimination that can have an effect on the professional identity formation and specialty choice. There are differences in personality among female and male students interested in surgery. Gender is a determining factor to choose surgery, with a consistent lower proportion of women compared interested in pursuing a surgical career. Mentoring and personality fit are important in medical student’s specialty selection. Female students are more likely to be discouraged from pursuing a surgical career by a lack of female role models.

**Conclusions:**

Bias against women in surgery still exists. There is a lack of studies that investigate the role of women in the teaching of surgery.

## Introduction

A considerable demographic shift has occurred and continues to occur in medicine as older physicians retire and a greater proportion of women enter the profession. The number of women entering medical schools today exceeds 50%, and the number in hospital specialties is expected to exceed 50% by 2016.^1^^,^^2^ As the gender ratio in the profession changes, the term “feminization of medicine”, referring to the medical profession becoming less dominated by men, has been a topic of debate in the medical literature.^3^ Women are increasingly entering the surgical profession,^4^ although the specialty is still male-dominated, with women representing 10-20% of the surgical workforce according to different studies.^2^^,^^5^ Also, the percentage of women as medical school faculty members holding associate or full professor rank remains well below the percentage of men.^6^ There is a global concern regarding the trend towards decreased interest in surgical careers: family considerations, increased stress and long work hours, sacrifice of personal time, and lack of (or negative) role models are the most common negative factors.^7^ In this context, very little is known of gender-related differences among medical educators in surgery at an undergraduate level. The apparent disparity in numbers and gender ratio relationship could possibly have some impact in students’ interest in pursuing a surgical career.

### Objectives

The overall objective with this review was to further the understanding regarding gender equality in surgical education to guide future initiatives. We aimed to explore the literature to ascertain whether there are gender differences in the teaching and learning of surgery, and if there are any gender differences when choosing and progressing in a career in surgery, and to explore the possible explanations to gender disparities within surgical education.

## Methods

### Focused questions

We aimed to explore evidence concerning gender differences in teaching and learning in surgery at an undergraduate level by answering the following questions:

1. Does gender influence the teaching of surgery? Is there any gender that is predominantly involved in teaching surgery for undergraduate medical students?
2. Does gender influence the learning process of surgery?
3. Is there any gender that predominantly is interested in surgical specialties?
4. If there are differences, how can they be explained?
5. We focused on studies that investigated gender differences in the teaching and learning of surgery in medical education. We applied the PICO terminology as described by Cook and West.^8^

### Search strategy

The search for relevant literature was performed in March 2013 by two investigators (CMB and AJ), who independently searched the following electronic databases: MEDLINE, EMBASE, CINAHL, PsycINFO, ERIC, Web of Science, Scopus and PubMed.The key search terms used included: gender, medical education, surgical education, surgery, undergraduate, academia, teaching and learning; and the following MeSH terms: education, medical, surgery, undergraduate, faculty, teaching, female, male, sex distribution, students.

### Inclusion and exclusion criteria

All studies related to gender differences in surgical education, teaching or learning of surgery, at an undergraduate level were included in the first stage. All articles not relevant for our purpose (gender differences in outcomes after surgical procedures, gender differences among professionals in surgical specialties) were excluded after reading the title (Step 1). After removal of duplicate records, all the titles and abstract were screened, and the final selection of articles was based on the following criteria (Step 2):

1. Published after 1990, and
2. Studies that focused on gender differences in academic surgery, or
3. Studies that focused on gender differences in the teaching of surgery at an undergraduate level, or
4. Studies that focused on gender differences in the learning of surgery at an undergraduate level, or
5. Studies that focused on gender differences in interest on surgical specialties

### Data abstraction/extraction

A standardized data extraction and critical appraisal instrument was constructed by the investigators using an electronic spreadsheet (Microsoft Office for Macintosh 2011) for the purpose of this review.

### Analysis

Discrepancies in opinions between the investigators were identified and final consensus was reached through discussion. The study methodologies were analyzed, and common themes from study findings were defined. Gender differences in learning were differentiated from gender differences in teaching. Gender differences in learning were differentiated in skills acquisition and perceptions and attitudes. Gender differences in interest on surgery were differentiated in factors influencing interest in surgical careers and personalities.

## Results

### Trial flow

**Figure 1:**
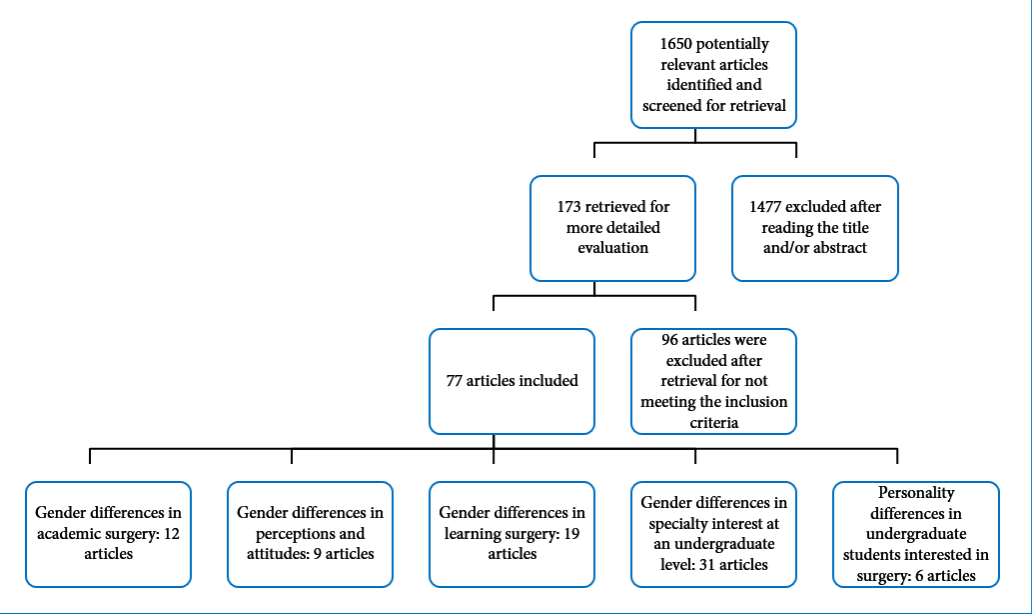
Trial flow

The literature search produced a total of 547 articles in the Web of Science, 705 in Scopus, 1,465 in PubMed, 127 in ERIC, 29 in Medline, 150 in EMBASE, 13 in PsycInfo and 58 in CINAHL, with a total 1,650 non-duplicated titles. After screening all titles, many were excluded for not being relevant for our purpose.After screening all titles and abstracts for potentially relevant articles, a total of 173 non-duplicated articles were obtained in full text for closer inspection, and of these 77 met the selection criteria (Figure 1).

### Study characteristics

The selected studies were classified in four categories:

#### I. Gender differences in academic surgery

There is a gross underrepresentation of women in the leadership positions of surgical departments. Both Flannery and Zhuge discuss the perception of a “glass ceiling”, a metaphor for the observation that how, despite the increasing numbers of female medical students, and increasing number of women entering surgical specialties, fields traditionally held by men, their advancement into the most prestigious, highest-paying, and leadership positions is still limited.^9^^,^^10^ Women in academic surgery seem to be less likely to be promoted than their comparably credentialed male colleagues.^10^^,^^11^ Despite the year of publication, the same pattern seems to persist according to a recently published survey, where the female participants, senior surgical residents and early career faculty members, experienced gender as a limitation to access a career in academic surgery.^6^

Similar results were reported by Sexton and colleagues when they examined the trends in gender-based advancement in academic surgery by performing a comparative analysis of the rate of change in the percentage of medical students, surgery residents, and full professors of surgery who are women, and concluded that percentage of full professors in surgery who are women is increasing at a rate disproportionately slower than the increases in female medical students and surgery residents.^12^Jonasson reviewed the number of women in the major surgical societies and organizations in US, and found that women are underrepresented, also in the leading positions of academic departments of surgery.^13^ Neither does the representation of women in professional societies and editorial boards reflect their presence in medical specialties, with an overrepresentation of men holding these positions (83%).^14^Despite being in minority, women are more likely to engage in clinical teaching. Klingensmith and Anderson performed a cross-sectional survey of membership of the Association for Surgical Education, and although the precise numbers of women in the US who are involved in educational scholarship is unknown, the numbers appear to be substantial; the percentage of women who have participated in the American College of Surgeons’ “Surgeons as Educators” course over the past 11 years of the course’s existence is 20.4%, and the number of women who have participated in the Association for Surgical Education’s “Surgical Education Research Fellowship” is 32% over the 10 years of its existence. These data suggest that women seek advanced training in educational scholarship in greater proportions than do men, given the proportions of female to male surgeons at large.^15^ Mendoza and colleagues also reported a majority of female educators, 58%, with doctoral degree and full-time faculty appointment in a surgery department in an American or Canadian institution.^16^ Although the number of participants in this study is low (only 12 respondents), the results are in accordance with those from the survey performed by Tesch and colleagues, where women are more likely to engage in clinical teaching (16.7% of female vs. 14.8% of male surgeons).^11^There are no significant differences between male and female junior surgeons involved in teaching undergraduates. They have similar attitudes toward, and practices in, voluntary undergraduate teaching.^17^ Prichard and colleagues performed a survey in an attempt to clarify whether there are differences between the attitudes and practices of male and female junior doctors regarding the practice of undergraduate teaching in the UK. With a majority of male doctors in surgical specialties (68.1%), there were no significant differences between the genders regarding the self-reported quantity of teaching provided to undergraduates. There are, however, differences in attitudes and personalities. Male doctors perceived themselves as more confident educators when compared to female doctors, but this could reflect cohort demographics in which a greater proportion of male doctors were more senior^17^ (Table 1).

**Table 1:** Gender differences in academic surgery

Author	Year	Participants	Type of study	Study objective	Findings
Cochran	2013	Senior surgical residents and early career surgical faculty members	Survey	To test the hypothesis than female surgeons perceive different barriers to academic careers relative to their male colleagues	Women experience gender as a barrier to develop a career in academic surgery.
Sexton	2012	Member of American Association of Medical Colleges	Cohort study	To examined the trends for gender-based advancement in academic surgery	The percentage of full professors in surgery who are women is increasing at a rate disproportionately slower than the increase in female medical students and surgery residents.
Prichard	2011	Non-consultant hospital doctors	Survey	To investigate differences in attitudes and practices of male and female junior doctors in the practice of undergraduate teaching.	No significant differences between the genders regarding the self-reported quantity of teaching provided to undergraduates. Male and female doctors have similar attitudes toward, and practices in, voluntary undergraduate teaching.
Zhuge	2011		Review	To analyze the factors that contribute to the glass ceiling phenomenon	Traditional gender roles, manifestations of sexism in the medical environment, and lack of effective mentors make women advance more slowly than men in academic surgery.
Morton	2007	Women and men on a professional society or journal editorial board	Survey	To quantify the number of women in this boards	Women's representation on societies and editorial boards does not always reflect their presence in medical specialties. 83% of board members were men, and occupied >80% of top leadership positions.
Hoover	2006		Editorial	To identify factors behind gender inequities in academic surgery	Underrepresentation of women at all levels of academic surgery; importance of mentoring.
Klingensmith	2006	Members of the Association for Surgical Education	Cohort study	To determine whether or not educational scholarship is a pathway to academic promotion	Overrepresentation of male in academic surgery (82 vs 18%). Women seek advanced training in educational scholarship in greater proportions than men. Academic promotion occurs for individuals with focus on education.
Mendoza	2004	Members of the Association of Surgical Education and the Association of Program directors in Surgery	Survey	To assess academic preparation and scholarly activity of educators in surgical departments	Professional educators provide support needed to meet the growing demands and requirements of surgical education. Predominance of women involved in education.
Risberg	2003	Physicians involved in medical teaching	Prospective cohort	To investigate gender awareness among physician teachers	There is an interaction between physician teachers' gender and specialty. Male physicians from the surgical group assessed gender less important than female physicians in professional relationship. No significant differences between specialty groups among women.
Flannery	2002		Review		Difficulties for women to progress in academic promotion. More women involved in clinical teaching.
Jonasson	2002	Members of several American surgical societies	Cohort study	To assess the status of women in leadership positions in surgery	Underrepresentation of women in the major American societies and organizations, and in higher levels of academic departments of surgery.
Tesch	1995	American physician, AAMC database	Survey	To explain the fact that the percentage of women members of medical school faculty with professor rank is lower than men	Women physician medical school faculty are promoted more slowly than equal qualified men.

#### II. Gender differences in skills acquisition among undergraduate medical students learning surgery

Lee and colleagues tried to identify variables associated with clinical clerkship grades in a large survey, and found that females tend to obtain lower grades in internal medicine and surgery, whereas males got lower grades in obstetrics/gynecology and psychiatry.^18^Male students are more exposed and perform surgical procedures significantly more often than female students, and working experience seems to enhance the surgical skills of medical students, but research experience may impair the learning of these procedures. The systematic use of logbooks seems to be useful for both male and female students.^19^When e-learning is used as a complement to traditional teaching methods in undergraduate surgical teaching, there are no significant gender differences in the utilization of the online program. Student utilization of the multimedia learning tool was not associated with improvements on final-year examination results.^20^^, ^^21^Lee and colleagues did not find objective differences in dexterity in students interested in surgical fields when compared with those interested in nonsurgical fields,^22^ whereas Elneel and colleagues found that right-handed males exhibited a greater level of ambidexterity than left-handed males and right-handed females, and more efficient task performance as measured by execution time.^23^The majority of the studies that investigate gender differences in surgical skills acquisition are related to laparoscopic skill acquisition in a simulator environment, and the results are contradictory. Some studies have shown that males tended to perform better than females in completing tasks that required the use of visual-spatial manipulation of the instruments within a simulated laparoscopic or endoscopic environment,^24^^-^^29^ whereas other studies have shown no difference in performance.^28^^,^^30^^-^^35^ Even though men completed the tasks in less time than women in some studies, there were no statistical difference between the genders in the number of errors and unnecessary movements.^29^ Despite that, women display initial lower performance, but respond to simulator training at least as well as men and reach parity with men’s performance after training^24^ (Table 2).

**Table 2:** Gender differences in skills acquisition of undergraduate medical students learning surgery

Author	Year	Participants	Number of participants male/female	Type of study	Study objective	Findings
White	2012	4th year medical students and 1st year residents	Students 80/45; Residents 40/16	Retrospective analysis of outcomes	To investigate gender difference in the acquisition of laparoscopic skills	Women display initial lower performance, but respond to training as well as men and reach parity with men’s performance after training.
Lee	2012	3rd year medical students	100 (59/41)	Prospective	To examine the impact of innate manual dexterity on the career interests of medical students	5 female interested in surgical fields vs 16 male. No objective difference in dexterity in students interested in surgical fields when compared with those interested in nonsurgical fields.
Thorson	2012	4th year medical students	16/16	Prospective	To investigate the performance in laparoscopy simulation of students interested in surgery, vs not, and differences by gender	11male/6 females interested in surgery. Female students have less innate abilities to laparoscopy.
Kolozsvari	2011	Medical students	32 (19/13)	Prospective, intervention study	To evaluate the impact of gender on the learning curve for a fundamental laparoscopic task	Gender did not affect the learning curve for a fundamental laparoscopic task, while interest in surgery and perceptual abilities did influence early performance.
Szczepanik	2010	Surgeons and medical students	17 surgeons 44 students	Prospective	To assess motor coordination skills in medical students, surgical residents and attending surgeons	No gender differences in coordination skills.
Adamczyk	2009	1st year medical students	850 (Unknown proportion male/female)	Survey and retrospective review of examination results	To investigate how students use multimedia tools to support their learning during a gross anatomy dissection course	45% of male and 33% of female students used the multimedia-learning tool, both groups found it equally valuable. No gender differences in performances. No significant correlation between the use of the multimedia learning tool and the exam performance of students.
Shane	2008	4th year medical students and 1st year residents	26 (17/9)	Prospective	To investigate if previous video game experience facilitates acquire new surgical skills	Previous video game experience shortens time to achieve proficiency on two tasks on a validated surgical simulator. Women perform worse than men.
Madan	2008	Preclinical medical students	51	Prospective	To investigate if baseline laparoscopic skills scores could be predicted in an inanimate box trainers and virtual-reality trainers	No gender differences in box trainer, male performed better in virtual-reality trainer.
Table 2 (Cont.)						
Author	Year	Participants	Number of participants male/female	Type of study	Study objective	Findings
Elneel	2008	Medical students	50	Prospective	To determine innate dexterity and ambidexterity across handedness and gender	Right-handed males exhibited a greater level of ambidexterity than left-handed males and right-handed females, and more-efficient task performance as measured by execution time.
Lee	2007	3rd and 4th year medical students	2395 (949/1328)	Survey	To identify variables associated with clinical clerkship grades	Lower grades for females in internal medicine and surgery, lower grades for males in ob/gyn and psychiatry.
Rosenthal	2006	Medical students	20 (12/8)	Prospective, intervention study	To evaluate the performance curves on repetitive trials for a laparoscopic task on a simulator, and the inter-individual differences after training	The performance curves for some of the simulator measurements were very close to logarithmic curves, and there were significant inter-individual differences in performance at the end of the repetitive trials. No significant gender differences observed.
Donnon	2005	1st and 2nd year medical students	42 (21/21)	Randomized control	To study the effect, between genders, of cognitive imaging as a teaching method in learning a surgical technique.	Males tended to perform better than females in completing tasks that required the use of visual-spatial manipulation of the instruments within a simulated laparoscopic environment.
Madan	2005	1st and 2nd year medical students	68	Prospective, intervention study	To explore if baseline laparoscopic surgery skills can be predicted.	No gender differences in laparoscopic skills. It is difficult to predict baseline laparoscopic surgery skills.
Healy	2005	Medical students	148 (48/90)	Prospective observational study	To evaluate the utilization of the web-based program	Electronic learning complements traditional teaching methods in undergraduate surgical teaching. No significant gender differences in the utilization of online access. Student utilization of the program was not associated with improvements on final year examination results.
Enochsoon	2004	Medical students	17(8/9)	Prospective, intervention study	To investigate factors that can predict performance in simulated gastroscopy	No significant gender-specific differences in the performance of the simulator, although there was a trend toward men performing better in two of the metrics, time of endoscopy, and efficiency of screening.
Grantcharov	2003	Novel residents	25 (18/7)	Prospective, intervention study	To identify factors influencing surgeons' performance in a computer simulator for laparoscopy	Men completed the tasks in less time than women, but there was no statistical difference between the genders in the number of errors and unnecessary movements.
Helenius	2002	Last year medical students	404 (138/266)	Survey	To gather information on the basic surgical skills of graduating medical students and to establish the factors influencing the students' competence	Final-year medical students have good theoretical knowledge of basic surgical procedures, but the successful performance rates range from 1 to 90%. Males performed surgical procedures significantly more often than females. Working experience enhanced the surgical skills, but research experience impaired the learning of the procedures. The systematic use of logbooks seems to be useful.
Mehrabi	2000	4th year medical students	103 (78/72)	Prospective, intervention study	To develop and evaluate a Computer Based Training (CBT) program for student	No gender difference in the outcome. CBT modules are an appropriate future teaching and learning system.

#### III. Gender differences in attitudes and perceptions among undergraduate medical students learning surgery

### Attitudes

Snelling and colleagues investigate how attitudes toward dissection vary with gender and ethnicity. Females were consistently more concerned about the physical aspects of dissection whereas there were fewer gender differences in the emotional responses.^36^

### Perceptions

Experiences of sexual harassment and gender discrimination have an effect on the professional identity formation and specialty choice of medical students. Female students choosing general surgery were those most likely to experience gender discrimination and sexual harassment during residency selection, and to be dissuaded from pursuing a career in that specialty.^37^^,^^38^ Female medical students do not feel equipped to respond to the unprofessional behavior of male supervisors, resulting in feelings of guilt and resignation over time that such events would be a part of their professional identity.^39^Lempp and Seale^40^ performed semi-structured interviews of 36 medical students throughout their medical education. Students did not report any gender-related differences in their training, although they used gender-related terms in their answers consistent with traditional gender stereotypes. A majority stated that surgery was dominated by men, reporting that the specialty required physical strength, competitiveness, unusually hard work and long working hours in order to succeed. Nevertheless, four female students (of 21) and four male students (of 15) were considering surgery as a career option. Most students (23/36) identified certain specialties as being ‘suitable’ for women, these being (in descending order of frequency) obstetrics and gynecology, general practice, pediatrics and palliative care. Eleven students (7 females, 4 males) used the word ‘sacrifice’ in relation to women and their medical career, for example in having to limit either their career or their family aspirations, but none used this remark to describe the careers of male doctors.^40^The perception of women medical students of the career satisfaction of women surgeons did not appear to be affected by the proportion of women surgeons on the faculty at their medical school. Their choice of surgery as a career was strongly associated with a higher proportion of women on the surgical faculty^41^ (Table 3).

**Table 3:** Gender differences in undergraduate medical students learning surgery: attitudes and perceptions

Author	Year	Participants	Number of participants male/female	Type of study	Study objective	Findings
Attitudes						
Jack	2010	Surgical faculty, residents, and medical students	61 Faculty 96 Residents 183 Medical students (90/93)	Survey	To assessed learning styles in medicine and to compare teaching and learning preferences	Medical students preferred converging learning (42%) and cluster 4 teaching (35%), no gender differences observed, no differences regarding specialty. 24% of students interested in surgery, no specification of gender.
Snelling	2003	1st year medical and dental students	467(173/294)	Survey	To investigate how attitudes toward dissection vary with gender and ethnicity	Females were consistently more concerned about the physical aspects of dissection whereas there were fewer gender differences in the emotional responses.
Perceptions						
Babaria	2012	3rd year medical students	12(0/12)	Interview	To investigate the spectrum of experiences of sexual harassment and gender discrimination and the effect on the professional identity of female students	Participants quickly learned how to confront and respond to inappropriate behavior. They did not feel equipped to respond to the unprofessional behavior of male supervisors, resulting in feelings of guilt and resignation over time that such events would be a part of their professional identity.
Lempp	2006	Medical students	36 (15/21)	Interview	To examine students' experiences and perceptions in relation to gender and ethnicity	No gender differences training experiences. Males experienced gender difficulties during the obstetrics and gynecology rotation. Students report gender-related distinctions consistent with traditional gender stereotypes.
Stratton	2005	4th year medical students	1027 (549/478)	Survey	To examine the role of gender discrimination and sexual harassment in medical students' choice of specialty and residency program	Women exposed indicated that gender discrimination and sexual harassment influenced their specialty choices. Men choosing obstetrics and gynecology experienced such behavior. Women choosing general surgery were most likely to experience gender discrimination and sexual harassment during residency selection.
Cochran	2003	3rd year medical students	93	Survey	To evaluate the impact of a general surgery clerkship on medical students’ perceptions of surgeons and surgical careers	Medical student perceptions of surgeons and surgical careers generally improve during the surgical clerkship, but students’ impressions of surgeons’ collegial behavior and commitment to teaching deteriorate significantly during the surgical clerkship.
Neumayer	2002	Final year female medical students	305 (0/305)	Survey	To identify if the proportion of women surgeons on the faculty influences the student's perceptions about women surgeons or their career choice	Women medical students’ perception of women surgeons' career satisfaction did not appear to be affected by the proportion of women surgeons on the faculty. Their choice of surgery as a career was strongly associated with a higher proportion of women on the surgical faculty.
Field	1996	1st and 5th year medical students	?	Interview	To investigate if gender affects future career choices and the ability to reach career goals	Females were seen as being disadvantaged both in terms of career choice and their ability to achieve career goals. Female students were more likely to suffer discrimination in specialties as surgery, and to be dissuaded from pursuing a career in that specialty.

#### IV. Gender and personality differences in undergraduate medical students’ interest in pursuing a career in surgery

Several of the studies have shown that gender has the greatest impact on specialty choice, with males choosing medicine, surgery, orthopedics and urology and females choosing obstetrics, pediatrics, anesthesiology, primary care and ophthalmology.^42^^-^^48^There is an overall declining popularity for surgical careers among both male and female students.^7^^,^^49^^-^^52^ On the other hand, other authors found general surgery to be the second preferred choice for men and the third preferred choice for women, with a total interest of 21% of the students.^43^Gender significantly influences the choice of surgery as a career, with a consistently lower proportion of women compared to men interested in pursuing a surgical career in different studies, in a range of 15-42% for male students and 2-29% for female students.^42^^,^^46^^,^^49^^,^^54^^-^^64^ Only two studies have shown that gender has no independent influence on choosing surgery as a career.^44^^,^^45^Male gender, updated preference, peer tutoring and selective training were the most significant predictors in the pathway to choosing surgery^46^ (Table 4).

**Table 4:** Gender differences in undergraduate medical students' career intentions in surgery

Author	Year	Participants	Number of articles/ participants male/female	Type of study	Study objective	Findings
Yu	2012	Medical students	17 articles	Review	To summarize the factors influencing female medical students when choosing a career in surgery	No gender differences in concerns about lifestyle and family priorities when considering a career in surgery. Gender discrimination and bias as a consistently and significant career deterrent reported by female medical students.
Fitzgerald	2012	Newly graduates	280 (78/130)	Survey	To investigate the perceptions of surgical careers among recent medical school graduates	42% males vs 25% females interested in a career in surgery. Reported negative attitudes toward women among the surgical teams. 59% of male and 68% of female respondents believed surgery was not a career welcoming women.
Bhat	2012	Interns/ Newly graduated	250 (130/120)	Survey	To investigate the factors influencing the preferences of medical graduates for specialization	Gender significantly influenced specialty choice with males choosing medicine and surgery and females choosing obstetrics and pediatrics. Factors which influenced specialty choice included job satisfaction, income, lifestyle friendliness and career prospects.
Zarebaczan	2011	3rd and 4th year students	505 (225/280)	Survey	To evaluate if the limitation of working hours increased medical students’ interest in pursuing a career in surgery	The 80-h work week has not improved medical students’ interests in surgery. 19% males vs 7.6% females were interested in surgery.
Maseghe Mwachaka	2010	Medical students	385	Survey	To assess specialty preferences and factors influencing specialty choices among male and female medical students	Significantly more males preferred surgery than females, who mainly selected pediatrics. Male students considered prestige in a specialty, female students considered ease of raising a family and gender distribution in the specialty.
Lefèvre	2010	6th year medical students	1742 (662/1080)	Survey	To analyze the aspirations and personal motivations behind the choice of surgical specialties	Gender as a determining factor to choose surgery: 44% of men vs 29% of women. Feminization, life style and income are the principal factors influencing the choice of the type of surgical specialization.
Deedar	2010	Medical students	39 articles	Review	To explore global trends related to medical student interest in surgical careers, and to identify factors influencing the choice of surgery as a career	Overall declining popularity of surgery, with the exception of Greece, Jordan, and Nigeria, and a relative rebound seen in US. Direct patient care, immediate intervention, and personal interest as common positive influential factors. Family considerations, increased stress and work hours, sacrifice of personal time, and lack of (or negative) role models as common negative factors.
Are	2009	4th year students	140 (67/73	Survey	To describe the attitudes of fourth-year medical students towards general surgery.	10% of students chose surgery (10 males and 5 females) Only 16% of the faculty department were female.
Table 4 (Cont.)						
Author	Year	Participants	Number of articles/ participants male/female	Type of study	Study objective	Findings
Maggiori	2009	Medical graduates	929 (291/638)	Cohort study	To analyze the choices of specialties of interns	21% interested in surgery, more male (41%) than female (22%).
Soethout	2008	1st-5th year students	3102 (1055/2047)	Survey	To investigate the association between biographical characteristics and academic achievement with preferences for specialty	Overall, more males interested in surgery than females. Medical background of the parents and gender are positively associated with a preference for a career.
Copmton	2008	Medical students	942 (501/441)	Survey	To describe patterns of change in specialty interests during medical school	Pediatrics (20%) and surgery (18%) the most common specialty choices among students. More women interested in primary care. Prestige more important for men than women (48% vs 36%).
Khader	2008	2nd, 4th and 6th year students	440 (287/158)	Cross-sectional study	To investigate the career preferences of medical students at Jordan University	Surgery as the preferred specialty by male students, obstetrics and gynecology by female students.
Scott	2008	1st year medical students	2168	Survey	To identify the interest in surgery and the factors that influence a student's interest in pursuing a surgical career	Declining preference for surgical careers among students, with only 21% (441, 238 males/203 females) of students interested. Male students interested in surgery were more hospital-oriented and less social orientated than female students.
Tambyraja	2008	Final year medical students	193 (75/118)	Survey	To examine the attitudes of students to surgical training and their career intentions.	53% of male students consider a career in surgery, 35% of female do.
Bamboolal	2007	1st year medical students	136 (58/78)	Survey	To determine factors that influence career choice among 1st-year medical students.	Declining preferences for surgical careers among males and females students: 19% of the students chose surgery (not specify by gender).
Nohr	2007	Medical graduates applying for residency	1920	Cohort study	To describe differences in specialty choices and qualifications between male and female physicians at the start of their residency in Denmark 1998-2003	Gender differences in specialty choice and in the way men and women qualify for residency: women achieved higher points for clinical qualification and theoretical courses, while men achieved higher points for scientific and pedagogical qualification. Most of residents in orthopedic surgery were male, in psychiatry or obstetrics female.
Buddeberg-Fischer	2006	Medical graduates	522 (241/281)	Prospective cohort	To investigate the influence of gender, personality traits, career motivation and life goal aspirations on the choice of medical specialty	Gender had the greatest impact on specialty and career choice. Males were more likely to choose a surgical career, females preferred obstetrics, pediatrics and anesthesiology.
Huda	2006	Final year students	232	Survey	To identify the career and gender preferences of the final year medical students	21% students chose surgery, general surgery being the 2nd preferred for men and 3rd preferred choice for women.
Sanfey	2006	Medical students	1300 (680/620)	Web-based survey	To investigate if the increased enrollment of female medical students and different priorities of the current generation of students would have influence on the declining interest in surgical careers	35% of women (3% men) were discouraged by a lack of female role models. More male than female students interested in surgery agreed that their skill sets were compatible with surgical careers (30% men vs 24% women). The decision to have a family had bigger influence for women than men, but family and lifestyle priorities were also important to male students.
Sobral	2006	Medical graduates	578 (318/260)	Cohort study	To examine the differential impact of person-based and program-related features on graduates' dichotomous choice between surgical or non-surgical specialties.	Declining preferences for surgical careers among males and females students: only 23% students choosing surgery, 26% of them female. Male sex, updated preference, peer tutoring and selective training were the most significant predictors in the pathway to choose surgery.
Table 4 (Cont.)						
Author	Year	Participants	Number of articles/ participants male/female	Type of study	Study objective	Findings
Harris	2005	Medical graduates	4259	Survey	To identify the relative importance of extrinsic determinants of doctors' choice of specialty.	Factors of particular importance to women, compared with men, were "appraisal of domestic circumstances", "hours of work" and "opportunity to work flexible hours".
Cochran	2005	4th year medical students	408 (214/189)	Cross-sectional internet survey	To analyze characteristics of general surgery residency and practice and their influence on student interest in surgical careers	Medical students who choose surgical careers are not deterred by a negative perception of lifestyle and workload considerations. Mentoring and personality fit are central in specialty selection. Surgery selected by 9%, with similar proportion male/female.
Ek	2005	Final year medical students	251 (126/125)	Survey	To determine the views of final year medical students in Melbourne regarding both undergraduate surgical teaching and the students' career aspirations.	Male students were more likely to be interested in surgery. 57% of female respondents felt discouraged from pursuing a surgical career and 99% of these women reported lifestyle/family factors as the main reason, with a further 72% identifying a lack of female role models.
Wendell	2003	Medical graduates	120 (48/54)	Survey	To evaluate factors affecting the choice of a surgical career among gender	Fewer women than men considered practice lifestyle in choosing their career. Both men and women considered lifestyle, elective in the field of choice and faculty important in career choice.
Azizzabeh	2003	4th year medical students	111 (48 M/ 31 F/ 32 unknown)	Survey	To determine factors that influence career choice among medical students	Prestige and career opportunities are more important to students seeking surgical residencies. Concerns about lifestyle and work hours are deterrents to surgery as a career. 19 (17%) students interested in surgery.
Figueireido	1997	Medical graduates	821	Data from Medical Faculty	To examine the influence of gender on the specialty choices of graduates from a Brazilian medical school during 2 periods	More male than female choosing surgery during both study periods (16 vs 2% resp 15 vs 6%).
Al-Faris	1997	5th (final) year medical students	253 (149/104)	Cross-sectional study	To identify the career choices and reasons for career choices of final-year Saudi medical students	16% students choose surgery. More men chose surgery, more women chose obstetrics-gynecology and ophthalmology.
Zulkifli	1997	5th (final) year medical students	241 (107/134)	Cross-sectional study	To investigate career choices among Malaysian medical students	Surgery ranked highest among male students, obstetrics and gynecology among female students.
Razali	1996	3rd year medical students	?	Survey	To investigate the reasons for entry to medicine and the career perspectives of phase III medical students of a Malaysian university	No significant differences in the intention of pursuing a career between genders, but women were less likely to seek entrance into private practice or pursue formal postgraduate education. The choice of surgery as a career was confined to men.
Baxter	1996	4th year medical students	245	Survey	To examine factors affecting career choice by medical students	Male more likely to choose a surgical career than females (27% versus 10%). Males were more likely to identity technical challenge, earning potential, and prestige whereas females were more likely to identify residency conditions, part-time work, and parental leave availability as important qualities in a specialty. Females were less likely to take surgical electives and more likely to identify a lack of role models.

Gender, prestige, career opportunities, direct patient care, immediate intervention, and personal interest are common positive influential factors for choosing a career in surgery.^7^^,^^47^ Gender discrimination, concerns about lifestyle, family considerations, increased stress and work hours and sacrifice of personal time are the most common negative factors to the pursuit of a career in surgery.^7^^,^^51^^, ^^54^Several authors have investigated the relationship between personality and specialty interest. Students interested in ‘surgical’ specialties obtain higher scores on ‘impulsive sensation seeking’ ‘aggression-hostility’ and ‘sociability’ scales and lower scores on a measure of ‘neuroticism-anxiety’. Male students had significantly higher scores on the ‘impulsive sensation seeking’, and surgery was the single most popular specialty among male students.^48^^,^^49^Coulston and colleagues found that significantly fewer females than males rated surgery highly likely as a career. They also found that females interested in surgery had higher neuroticism and agreeableness scores, and placed greater importance on the ability to help people, and less importance on prestige and financial reward compared to males interested in surgery. Compared to males not interested in surgery, females interested in surgery had higher Openness scores, and placed greater importance on ability to help people, interesting and challenging work, and less importance on lifestyle. Also, females interested in surgery had lower Agreeableness scores, and placed greater importance on prestige and less importance on lifestyle compared to females not interested in surgery. Common findings were that surgeons compared to non- surgeons are more tough-minded, less patient-oriented and less empathic, and may be a function of the prevalence of males in surgery.^50^Professional attitudes in particular patient centeredness were shown by Batenburg and colleagues to be related to specialty preference in the final year of graduate medical training and specialty as a career choice: general practice trainees showed more patient-centeredness than surgery trainees. Gender was not related to patient-centeredness.^51^Significant gender differences were found when comparing empathy levels in medical students at different levels of education, on “emotional intelligence”, “empathy” and the “Utilization of Emotion” subscale, with females scoring higher than males on all three scales. There were no significant gender differences in end-of-year marks in any of the 3-year groups, with male empathy scores increasing between years 1 and 2, while female scores declined.^52^Frantsve and colleagues examined the effects of the applicant’s personality and gender on faculty rankings and matching to surgical residency program. Male applicants were more likely than female applicants to enjoy teamwork and assume a submissive role when interacting with authority figures. The faculty interviewers perceived female applicants as less likely to effectively cope with stress. They also found that female applicants might be less likely than males to be as friendly and deferent in their interactions with male authority figures. When compared to the general population, however, female applicants did not demonstrate evidence of difficulties with stress management^53^ (Table 5).

**Table 5:** Personality differences in undergraduate students interested in surgery

Author	Year	Participants	Number of participants male/female	Type of study	Study objective	Findings
Mehmood	2013	Medical students	546 (336/218)	Survey	To study the influence of gender and the personality profiles of medical students in their specialist choices	Surgery was the single most popular specialty amongst both male and female students. Males had significantly higher scores on the ‘impulsive sensation seeking’ scale and students preferring a surgery specialty had the highest score on the ‘impulsive sensation seeking’, ‘neuroticism-anxiety’, ‘aggression-hostility’ and ‘sociability’ scales.
Coulston	2012	2nd year medical students	573(267/304)	Survey	To study personality of female medical students attracted to the surgical profession.	Significantly fewer females rated surgery highly likely as a career. Females interested in surgery had higher Neuroticism and Agreeableness scores, and placed greater importance on ability to help people, and less importance on prestige and financial reward. Compared to males not interested in surgery, females interested in surgery had higher Openness scores, and placed greater importance on ability to help people, interesting and challenging work, and less importance on lifestyle. Females interested in surgery had lower Agreeableness scores, and placed greater importance on prestige and less importance on lifestyle compared to females not interested in surgery.
Hojat	2008	Medical students	1076 (517/559)	Survey	To investigate the relationship between personality and specialty interest	Students interested in ‘surgical’ specialties obtain higher scores on a measure of ‘impulsive sensation seeking’ and lower scores on a measure of ‘neuroticism-anxiety’. Students interested in ‘hospital-based’ specialties score lower on a measure of ‘sociability’ whereas those interested in ‘primary care’ score higher on this measure. Results also showed that men scored higher on ‘impulsive sensation seeking,’ and women outscored men in the ‘neuroticism–anxiety’ and ‘activity’ scales. More male interested in surgery.
Austin	2007	Medical students 2nd, 3rd and 5th year	273 (85/188)	Survey	To examine gender differences in empathy levels in medical students at different levels of education, and to investigate whether emotional intelligence and empathy are related to academic success	Significant gender differences were found for the whole sample on emotional intelligence, empathy and the Utilization of Emotion subscale, with females scoring higher than males on all 3 scales. There were no significant gender differences in end-of-year marks in any of the 3 year groups, with male empathy scores increasing between years 1 and 2, whilst female scores declined.
Frantsve	2002	Applicants to a OMS residency program	48 (38/9)	Interview	To examine effects of applicants’ personality and gender on faculty rankings and matching to an Oral and Maxillofacial Surgery residency program	Male applicants enjoy more teamwork and assume a submissive role when interacting with authority figures, female applicants less likely than males to be as friendly and deferent in their interactions with male authority figures.
Batenburg	1999	Final year medical students	40(16/24)	Survey	To asses patient-centeredness of trainees in general practice and surgery and of final-year clerks preferring one of these specialties	Professional attitudes, in particular patient-centeredness, seem to be related to specialty preference in the final year of graduate medical training and specialty as a career choice: general practice trainees showed more patient-centeredness than surgery trainees. Gender was not related to patient-centeredness.

## Discussion

Despite the dramatically increased entry of women into surgery, a traditionally male-dominated field, there remains a gross underrepresentation of women in leadership positions of these departments.^10^ Female surgeons perceive gender as a limitation to pursuing a career in academic surgery,^6^^,^^9^^-^^11^ this is not only a perception, since women are underrepresented in the major surgical societies, organizations and leading positions of academic departments of surgery.^12^^-^^14^ However, female surgeons seem to be more actively involved in teaching undergraduates,^11^^,^^15^^,^^16^ but there is an overall lack in the literature of recent studies that investigate this observation.As Zhuge and colleagues pointed out, the major constraints of contributing to the glass-ceiling phenomenon are traditional gender roles, manifestations of sexism in the medical environment, and lack of effective mentors.^9^ Gender roles contribute to unconscious assumptions that negatively influence decision-making when it comes to promotions. The lack of role models in surgery in general, and academic surgery in particular, has been discussed in different articles as a possible explanation for the underrepresentation of women in surgical academia.^9^^,^^54^^,^^55^ Mentoring women to success in academic surgery, and identifying barriers to women entering surgery and achieving positions in academic surgery are prerequisites for correcting the existing gender inequities.Sexism has many forms, from subtle to explicit forms, and some studies show that more women report being discriminated than men. Gender discrimination and bias has been reported as a consistently and significant career deterrent reported by female medical students.^56^Female students pursuing a surgical career have experienced discrimination,^37^^,^^38^^,^^57^^,^^53^ although this is not an isolated phenomenon for the specialty: female students interested in a career in orthopedic trauma, or male students interested in obstetrics had such experiences.^37^^,^^38^^, ^^58^^, ^^59^It is not clear if gender itself is a factor that affects the learning of fundamental surgical skills.^24^^-^^35^ Work experience, interest in surgery, training and previous video game experience seem to enhance the surgical skills of medical students.^19^^,^^24^^,^^26^^,^^28^^,^^30^ Greater exposure to surgery and previous experience in video games among male students could be some of the explanations for the better performance observed in various studies measuring laparoscopic skills, even though training seems to level out those initially observed differences. The overall decreased interest among female students in surgery,^22^^,^^25^ could be a possible explanation for women’s poorer early performance.^30^But it seems clear that gender is a determining factor for choosing surgery, with a consistently lower proportion of women compared to men interested in pursuing a surgical career (15-42% males vs. 2-29% females).^42^^,^^46^^,^^49^^,^^54^^-^^64^ The choice by females of surgery as a career is strongly associated with a higher proportion of women on the surgical faculty,^41^ again highlighting the importance of role models and mentoring.^10^^, ^^60^^-^^62^

### Limitations

The majority of the authors of the reviewed articles are women, and this can be a potential source of bias. Most of the articles that investigate students’ attitudes, perceptions, personality and interest for surgery are surveys, where the students are self-selected for each study, itself representing a limitation of the study and thus being a source of potential bias. The intervention studies were done with the purpose of setting up baseline laparoscopic skills or to demonstrate the hypothesis that training laparoscopic surgery in a simulation environment improves performance. As the majority of the studies come from North America, the results may not apply to other countries. There are, however, some studies from other countries with consistent overall results.

### Implications

Gender equality, defined as the absence of discrimination on the basis of a person’s gender in opportunities, is not a spontaneous process. However, gender differences must first be identified and the imbalance between the genders should be addressed and rectified. There are known differences between male and female physicians, and gender also plays a role when it comes to choosing a specialty.^63^^,^^64^ To highlight these differences and the apparent gender inequalities is the first step of working towards gender equality within surgical education.Choice of surgery as a career by women is strongly associated with a higher proportion of women in the surgical faculty.^41^ The negative loop of few female role models in academic surgery that results in few female students interested in surgery will hardly change the trend of gender inequalities in surgery.Many have postulated that women are deterred from a surgical career because it continues to have an aura of being an ‘‘old boys’ club’’, and unfortunately, this review supports that statement. Bias against women in the field of surgery exists, and is a reality in the 21^st^ century.

## 

### Conflict of Interest

The authors declare that they have no conflict of interest.
